# Influence of Polymer Backbone Architecture and Amino Group Density on the Cytotoxicity and Transfection Performance of Cationic Block Copolymer Polyplexes

**DOI:** 10.3390/polym18212671

**Published:** 2026-10-28

**Authors:** Nikita A. Kozyrev, Alexey V. Doroshchenko, Anna D. Lapanik, Natalia L. Klyachko, Anton V. Lopukhov

**Affiliations:** 1 Laboratory for Chemical Design of Bionanomaterials, Faculty of Chemistry, M.V. Lomonosov Moscow State University, 117234 Moscow, Russia; n.kozyrev99@gmail.com (N.A.K.); alexey07251@gmail.com (A.V.D.); anya73anya@gmail.com (A.D.L.); 2 Faculty of Chemistry, Shenzhen MSU-BIT University, Shenzhen 518100, China

**Keywords:** cationic block copolymer, dopamine, targeted gene delivery

## Abstract

The development of efficient and biocompatible polymeric vectors remains a cornerstone of successful gene therapy. In this study, we developed a versatile polymeric platform for nucleic acid delivery by synthesizing a series of cationic block copolymers based on polyaspartamide or polyglutamide backbones and polyethylene glycol (PEG) segments. Block copolymer precursors were synthesized via ring-opening polymerization. The degrees of polymerization were confirmed via ^1^H NMR spectroscopy, followed by functionalization with diethylenetriamine, spermine, or spermidine. Cytotoxicity was evaluated using the WST-1 assay in HEK293 and SH-SY5Y cells. Cytotoxicity profiling identified N_3_-PEG_114_-b-pAsp(DET)_62_ as the least cytotoxic polymer among the tested derivatives in both cell lines. The polyplexes enhanced reporter gene expression compared with naked plasmid DNA, and an N/P ratio of 15 was selected for subsequent comparative experiments. In SH-SY5Y neuroblastoma cells, the material obtained following the dopamine-modification procedure exhibited a 1.78-fold increase in reporter fluorescence and a 1.75-fold increase in the percentage of GFP-positive cells compared with the corresponding unmodified formulation. These findings establish the engineered block copolymers as highly adaptable, low-toxicity, and efficient candidates for targeted nucleic acid delivery and support the potential of dopamine functionalization for enhancing delivery to dopamine-receptor-expressing cells.

## 1. Introduction

Neurodegenerative diseases represent a vast spectrum of severe disorders characterized by progressive structural damage and functional impairment of central nervous system neurons. Prominent among these conditions are Alzheimer’s, Parkinson’s, and Huntington’s diseases, alongside amyotrophic lateral sclerosis. To date, managing these pathologies has remained a formidable challenge for modern medicine. However, the emerging accessibility of high-throughput sequencing has fundamentally transformed diagnostic capabilities, enabling early-stage detection and timely therapeutic intervention. Furthermore, the evolution of gene therapy introduces the possibility of addressing the root causes of these diseases, thereby paving the way for more efficacious therapeutic strategies [1,2,3].

Gene therapy represents a promising therapeutic paradigm centered on the introduction of exogenous genetic material for disease prevention and treatment. Current methodologies involve several distinct strategies, such as the transport of genes mediating protective effects, the substitution of a defective gene with a functional copy, and the inactivation of malfunctioning genes [4,5]. However, the effective delivery of nucleic acids is inherently constrained by their intrinsic structural and physicochemical properties, as well as susceptibility to enzymatic degradation by extracellular and intracellular exo- and endonucleases, alongside other biological matrix components. Furthermore, successful translation requires navigating formidable biological barriers with distinct physiological properties, most notably the cellular membrane and the blood–brain barrier [6,7].

To overcome these limitations, specialized vectors engineered for nucleic acid delivery have been developed, among which polymers represent a major class. Polymeric vehicles facilitate efficient condensation of nucleic acids and, in contrast to viral counterparts, exhibit a significantly lower risk of immunogenicity and mutagenesis. Another prominent benefit of polymeric carriers is the high versatility of their physicochemical properties, which can be precisely modulated via the selection of different polymer backbones and various chemical functionalization techniques [8,9]. In particular, cationic polymers are widely utilized for transfection owing to the electrostatic interactions between their positively charged moieties and the negatively charged phosphate backbone of nucleic acids, as well as anionic cellular membranes. However, polymeric formulation strategies still face critical bottlenecks, including relatively low delivery efficiency and elevated cytotoxicity [10].

From a structural perspective, polymers can be classified into several distinct categories, including natural polymers, polypeptides, dendrimers, and other specialized entities like polyethylenimine [11]. Another widely accepted classification is based on their degradation profiles, distinguishing between non-biodegradable polymers, biodegradable natural polymers, and biodegradable synthetic polymers. Specifically, polyamidoamines fall into the first category, chitosan and β-cyclodextrins comprise the second, and polylysine (PLL) represents the latter category. The major assets of biodegradable vehicles include superior biocompatibility, bioactivity, and predictable biodegradability. In contrast, a lack of such properties severely restricts the development and application of non-degradable synthetic alternatives. Currently, biodegradable synthetic polymers are predominantly fabricated utilizing two synthetic approaches: step-growth polymerization and ring-opening polymerization [12].

Polyaspartamide constitutes a prominent representative of synthetic polymers obtained through the ring-opening polymerization of carboxyanhydrides. The primary advantage of polyaspartamide-derived carriers resides in their high biodegradability, which consequently ensures low cytotoxicity toward cell cultures even at high concentrations [13]. Within the domain of non-viral gene delivery, PEGylation represents a widely adopted functionalization approach. The integration of PEG segments into the polymer backbone or into liposomal formulations can increase the stability of polymer/DNA complexes (polyplexes), extend blood circulation lifetime, reduce cytotoxicity, and safeguard the complexes against opsonization and phagocytosis; however, in certain contexts, PEGylation may concurrently diminish gene delivery efficiency [14,15,16].

Cationic side chains containing protonatable amino groups facilitate electrostatic interactions with nucleic acids and may influence their intracellular delivery [17]. The buffering properties of such groups have been proposed to promote endosomal escape through the so-called proton sponge effect. However, the contribution of this mechanism remains debated, and alternative processes, including direct membrane destabilization, may also be involved [18,19,20]. Therefore, the incorporation of diethylenetriamine, spermine, or spermidine, which possess the intrinsic ability to efficiently condense DNA, should be considered a strategy for modulating the physicochemical and biological properties of polymeric carriers rather than evidence of a specific intracellular delivery mechanism [21,22].

PEG–polyaspartamide-based copolymers have been extensively investigated as non-viral vectors for nucleic acid delivery. In particular, PEG-pAsp(DET) polyplexes have demonstrated efficient plasmid DNA delivery in both *in vitro* and *in vivo* models. Previous studies have also explored the incorporation of polyamine-containing side chains and targeting ligands to modify the biological performance of these carriers [23,24,25].

The potential advantages of polyaspartamide-based carriers include their structural versatility and, in certain derivatives, their susceptibility to degradation under physiological conditions. For example, previous work demonstrated that pAsp(DET) undergoes self-catalytic degradation, which was associated with reduced cumulative toxicity compared with a non-degradable polyglutamide analogue [13]. Nevertheless, biodegradability alone does not guarantee low cytotoxicity, which also depends on polymer composition, cationic side-chain structure, concentration, and interactions with cellular membranes.

Against this background, the present study comparatively evaluates PEG-based polyaspartamide and polyglutamide derivatives bearing different cationic side chains, with particular emphasis on their cytotoxicity and functional plasmid DNA delivery in HEK 293 and SH-SY5Y cells.

Among possible ligands, dopamine has attracted attention because of its biological relevance to neuronal cells and the expression of dopamine receptors in several neuronal cell models [26]. Previous studies have demonstrated that dopamine-functionalized delivery systems can exhibit enhanced interactions with neuronal cells. Previous work demonstrated increased uptake of dopamine-conjugated extracellular vesicles in SH-SY5Y cells and primary neurons [27]. In that study, competition with free dopamine and receptor-blocking experiments supported the involvement of dopamine receptors in the observed uptake. Although these findings provide a rationale for exploring dopamine functionalization in other delivery platforms, receptor recognition cannot be assumed to be preserved following dopamine conjugation, as both the amino and catechol groups contribute to dopamine–receptor interactions [28]. Furthermore, the effects observed for extracellular vesicles cannot be directly extrapolated to synthetic polymeric carriers.

Inspired by these advancements, herein we report the development of nucleic acid delivery systems based on polyaspartamide vectors modified with diverse aliphatic amines and the investigation of whether dopamine functionalization of the selected PEG–polyaspartamide carrier could enhance functional plasmid DNA delivery in SH-SY5Y neuroblastoma cells. This approach was intended to evaluate the potential of dopamine modification to improve transfection performance, without assuming a specific receptor-mediated uptake mechanism.

## 2. Materials and Methods

### 2.1. Materials

#### 2.1.1. Reagents

Methoxy(polyethylene glycol) amine (mPEG-NH_2_) (Mw 5000 g/mol), azide-functionalized poly(ethylene glycol) amine (N_3_-PEG-NH_2_) (Mw 5000 g/mol), benzyl (4S)-2,5-dioxooxazolidine-4-acetate 98% (BLA-NCA), benzyl (4S)-2,5-dioxooxazolidine-4-propionate 97% (BLG-NCA), and diethylenetriamine 99% (DET) were purchased from Aladdin (Shanghai, China). Dimethyl sulfoxide (DMSO) 99.9%, diethyl ether, hydrochloric acid 37%, 4-(2-hydroxyethyl)-1-piperazineethanesulfonic acid 99.5% (HEPES), and dopamine hydrochloride were purchased from Sigma-Aldrich (St. Louis, MO, USA). Acetic acid (99.8%), N,N-dimethylformamide (DMF), and chloroform (99.95%) were purchased from Component-Reaktiv (Moscow, Russia). Methoxy(polyethylene glycol)-block-poly-L-lysine (mPEG_114_-PLL_50_) (Mw 13000 g/mol) was purchased from Alamanda Polymers (Huntsville, AL, USA). Deuterated solvents CDCl_3_ (99.8%), DMSO-d6 (99.8%), and D_2_O (99.8%) were purchased from Solvex (Moscow, Russia). Dulbecco’s modified Eagle medium with Nutrient Mixture F-12 (DMEM/F12), pyruvate, L-glutamate, penicillin/streptomycin (P/S), trypsin–ethylenediaminetetraacetic acid solution (trypsin–EDTA), and fetal bovine serum (FBS) were purchased from Gibco (Paisley, UK).

#### 2.1.2. Plasmids

The pTurboGFP-C plasmid, a vector for TurboGFP expression in mammalian cells under the control of the CMV promoter, and the pTurboRFP-C plasmid, a vector for TurboRFP expression in mammalian cells under the control of the CMV promoter, were purchased from Evrogen (Moscow, Russia).

#### 2.1.3. Cells

Human embryonic kidney (HEK293, ATCC CRL-1573) and human neuroblastoma (SH-SY5Y, ATCC CRL-2266) cell lines were utilized in this study. Both cell lines were maintained in DMEM/F12 supplemented with 10% fetal bovine serum (FBS), 4.5 g/L glucose, L-glutamine, and an antibiotic/antimycotic cocktail (Gibco, UK). Cells were cultured in standard tissue culture-treated plasticware and incubated at 37 °C in a humidified atmosphere containing 5% CO_2_.

### 2.2. Synthesis of Block Copolymers

#### 2.2.1. Synthesis of PEG-b-pBLA and PEG-b-pBLG Block Copolymers

BLA-NCA or BLG-NCA (3.3 mmol) was dissolved in DMF (1.36 mL), followed by the addition of chloroform (6.8 mL). Concurrently, mPEG-NH_2_ (Mw 5 kDa) or N_3_-PEG-NH_2_ (Mw 5 kDa) (0.06 mmol) was dissolved in anhydrous chloroform (3 mL) and introduced into the BLA-NCA solution. The resulting reaction mixture was stirred at 40 °C under an argon atmosphere for 24 h. Afterward, the mixture was precipitated into a 10-fold volume of ice-cold diethyl ether (0 °C). The resulting precipitate was collected via filtration under reduced pressure, washed thoroughly with diethyl ether, and subsequently dried in vacuo. The reaction yields were 0.89 g (84%) for N_3_-PEG_114_-b-pBLA_62_ and 0.80 g (78%) for mPEG_114_-b-pBLG_55_.

#### 2.2.2. Synthesis of PEG-b-pAsp(DET) and PEG-b-pGlu(DET) Block Copolymers

The block copolymers N_3_-PEG_114_-b-pBLA_62_ or mPEG_114_-b-pBLG_55_ (19.6 umol) were dissolved in DMF (10 mL). Subsequently, DET (50 equivalents relative to the benzyl groups of the PBLA/PBLG segment) was introduced under anhydrous conditions at 40 °C. The reaction mixture was stirred for 24 h. Afterward, the mixture was added dropwise into an aqueous acetic acid solution (10% *v*/*v*, 40 mL) and subjected to dialysis against 0.01 N HCl utilizing a 6 kDa molecular weight cut-off (MWCO) membrane. The resulting product was lyophilized and stored at 0 °C under an argon atmosphere. The obtained yields were 0.32 g (94%) for N_3_-PEG_114_-b-pAsp(DET)_62_ and 0.22 g (67%) for mPEG-b-pGlu(DET)_55_, respectively.

#### 2.2.3. Synthesis of PEG-b-pAsp(spn), PEG-b-pAsp(spd), and PEG-b-pGlu(spd) Block Copolymers

N_3_-PEG_114_-b-pBLA_62_ or mPEG_114_-b-pBLG_55_ block copolymers (5.64 µmol) were dissolved in DMF (10 mL). Spermine or spermidine (50 equivalents of the benzyl group of the polymer segment) was dissolved in 50 mL of DMSO and then added under anhydrous conditions at 40 °C. The reaction mixture was stirred for 24 h. The mixture was added dropwise to a 10% *v*/*v* acetic acid solution (200 mL) and then dialyzed against 0.01 N HCl using a membrane with a molecular weight cut-off (MWCO) of 6 kDa. The product was dried via lyophilization and stored at 0 °C in an argon atmosphere. The yields ranged from 80% to 90%.

#### 2.2.4. Synthesis of Dopamine-Targeted PEG-b-pAsp(DET) Block Copolymer Conjugate

Propargyl-PEG_5_-NHS (0.023 g) was dissolved in 1.5 mL of 10 mM HEPES buffer solution (pH 7.4), followed by the addition of dopamine (0.109 g, 10-fold molar excess). The reaction mixture was incubated at 4 °C for 24 h. Subsequently, N_3_-PEG_114_-b-pAsp(DET)_62_ (0.1 g), pre-dissolved in 1.5 mL of 10 mM HEPES buffer solution (pH 7.4), was introduced into the resulting solution, along with 3 µL of a catalyst mixture containing 10 mM CuSO_4_·5H_2_O and 50 mM sodium ascorbate. The reaction mixture was stirred periodically for 1 h and then kept at 4 °C for 24 h. To purify the conjugate, the product was dialyzed against 0.01 N HCl utilizing a 6 kDa molecular weight cut-off (MWCO) membrane. The residual concentrations of free dopamine and copper in the purified material were not determined analytically.

### 2.3. Polymer Characterization

^1^H-NMR spectra were acquired using a Bruker 400 spectrometer at 25 °C. Dry polymer (~30 mg) was dissolved in CDCl_3_, DMSO-d_6_, or D_2_O (~500 µL). The spectra were calibrated using the solvent signals (CHCl_3_: 7.24 ppm, DMSO: 2.50 ppm, H_2_O: 4.80 ppm).

The nominal molecular masses of the synthesized polymers were estimated from the molecular mass of the PEG block and the degrees of polymerization determined by ^1^H NMR spectroscopy for the protected precursors. The calculations assumed quantitative substitution of the benzyl ester groups during aminolysis. These values represent theoretical estimates and should not be regarded as experimentally determined molecular masses or as independent confirmation of complete substitution. Polymer concentrations expressed in µM were calculated using the nominal molecular masses.

### 2.4. Polyplex Formation

The N/P ratio was operationally defined as the molar ratio of free primary amino groups in the polymer to phosphate groups in plasmid pDNA (pTurboGFP-c, 4701 bp, 1.82*10^15^ P/ug or pTurboRFP-c, 4713 bp, 1.82 × 10^15^ P/ug). For the nominal calculations, one free primary amino group per fully substituted repeating unit was assumed. The contributions of secondary amino groups and the actual degree of protonation were not included. Consequently, equivalent nominal N/P ratios do not necessarily correspond to equivalent effective cationic charge densities for polymers bearing different polyamine substituents.

To form the polyplexes, a solution of 1 µg pDNA in 150 µL serum-free media and different amounts of polymer in SFM were mixed with various precalculated N/P ratios up to 221 µL of total volume, immediately vortexed for 20 s, and then kept still for 30 min for further experiments. Hydrodynamic diameter (the intensity-mean Z-average diameter) and ζ-potential of the polyplex particles were determined using a Malvern Zetasizer instrument (Malvern Instruments Ltd., Malvern, UK) as previously described [29].

### 2.5. In Vitro Cytotoxicity Assay of Block Copolymers

The cytotoxicity of the synthesized polymers was evaluated by quantifying the activity of mitochondrial reductases in viable cells using the WST-1 assay. Briefly, HEK293 and SH-SY5Y cells were seeded into 96-well plates at a density of 2 × 10^4^ cells/well and cultured for 24 h in their respective growth media. Subsequently, the medium was replaced with a polymer solution diluted in culture medium to specific concentrations, followed by incubation at 37 °C under a 5% CO_2_ atmosphere. After 24 h of exposure, the treatment medium in each well was exchanged for fresh medium supplemented with 10% WST-1 reagent, and the cells were incubated for an additional 1 h. Material toxicity was determined based on the optical density of the solution, measured at a wavelength of 450 nm using a SpectraMax M5 microplate reader (Molecular Devices, San Jose, CA, USA). All experiments were performed in triplicate. All data were processed using Origin 7.0 software (OriginLab Corporation, Northampton, MA, USA). The final results are reported as the mean ± SD of three estimated IC_50_ values obtained from curve fitting using the Sigmoidal function (logistic function).

### 2.6. In Vitro Gene Delivery

To evaluate in vitro transfection efficiency, HEK293 and SH-SY5Y cells were seeded into 24-well plates at a density of 10^5^ cells/well and cultured for 24 h in antibiotic-free growth medium. Subsequently, the medium was replaced with serum-free medium, and polyplexes containing 1 µg of pDNA (pTurboGFP-C or pTurboRFP-C) at various N/P ratios were introduced, followed by incubation for 24 h at 37 °C. The transfection medium was then exchanged for complete growth medium, and the cells were incubated for an additional 24 h.

Transfection outcomes were assessed using three complementary approaches. Qualitatively, GFP expression was visualized via fluorescence microscopy (Nikon Eclipse Ti), monitoring the signal at λ_ex_ = 482 nm and λ_em_ = 502 nm.

For quantitative evaluation, cells were treated with a lysis buffer for 20 min at 0 °C. The fluorescence intensities of GFP or RFP within the resulting lysates were quantified at λ_ex_ = 482 nm/λ_em_ = 502 nm or λ_ex_ = 553 nm/λ_em_ = 574 nm, respectively. These fluorescence data were subsequently normalized to the total protein content, as determined by the bicinchoninic acid (BCA) assay.

Alternatively, transfection efficiency was analyzed via flow cytometry. After incubation in complete medium, cells were harvested using 0.05% trypsin-EDTA, washed, and resuspended in Dulbecco’s phosphate-buffered saline (DPBS). Following centrifugation, the cell pellet was resuspended in the same buffer. The percentage of GFP-expressing cells was determined using a Navios flow cytometer (Beckman Coulter, Brea, CA, USA) equipped with a 488 nm laser and an FL-1 filter (525/50). The results are expressed as the percentage of GFP-positive cells.

All data were processed using Origin 7.0 software (OriginLab Corporation, Northampton, MA, USA). All experiments were performed in triplicate. Statistical significance was evaluated using one-way analysis of variance (One-Way ANOVA) followed by Dunnett’s post hoc test for multiple comparisons against the control group.

## 3. Results

### 3.1. Synthesis of Block Copolymers

**Scheme 1:**
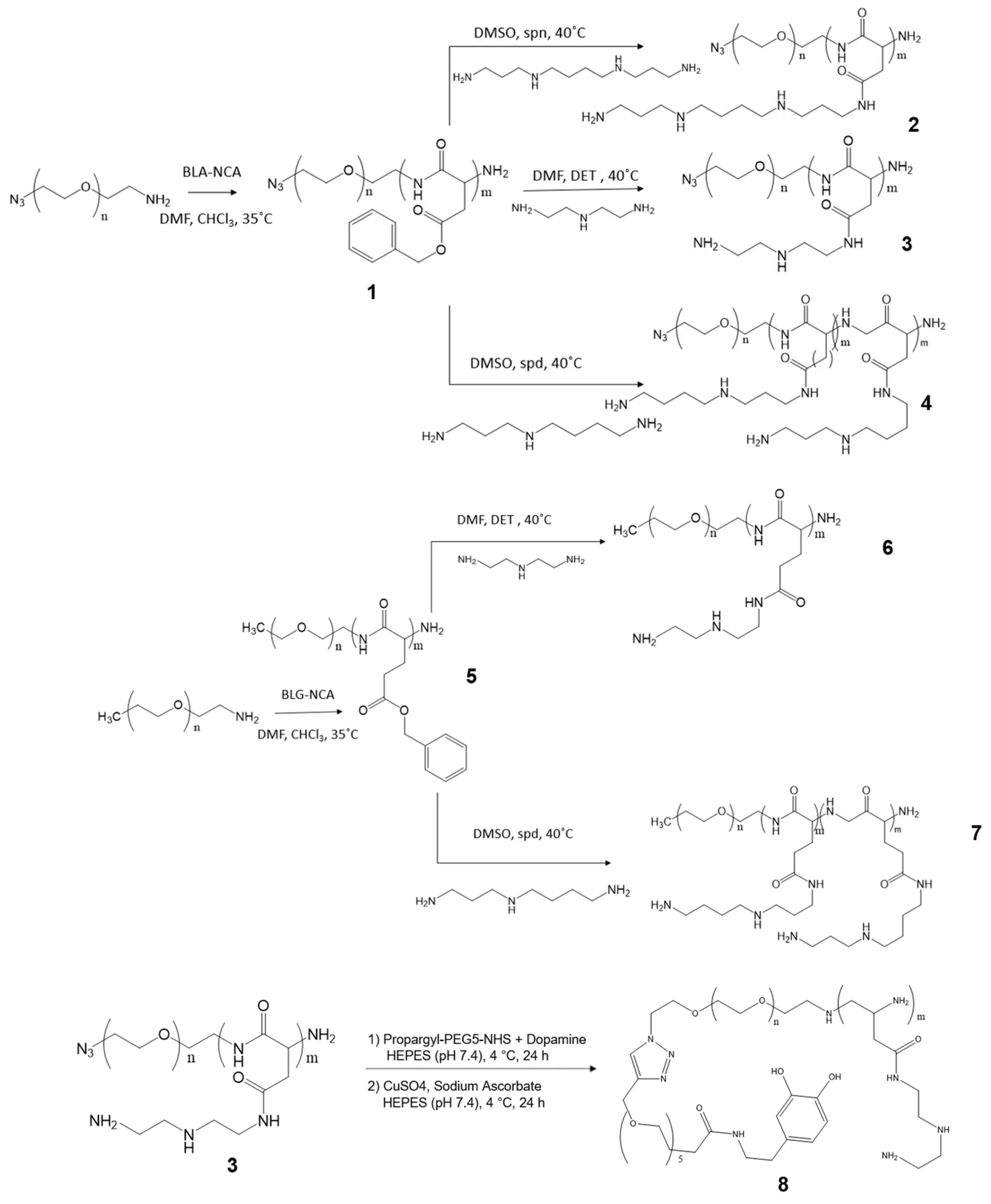
Synthesis of PEG-polyaspartamide and PEG-polyglutamide block copolymers with various cationic block structures. Spn—spermine; DET—diethylenetriamine; Spd—spermidine.

Cationic block copolymers featuring distinct architectures were synthesized via a two-step procedure. In the initial step, block copolymers comprising a polyethylene glycol block and a protected poly(aspartic acid) **1** or poly(glutamic acid) **5** segment were prepared (Scheme 1). The reaction proceeded through the ring-opening polymerization of the N-carboxyanhydride derivative of the corresponding amino acid, utilizing mPEG-NH_2_ (Mw 5 kDa) and N_3_-PEG-NH_2_ (Mw 5 kDa) macroinitiators.

The degree of polymerization was determined via ^1^H NMR spectroscopy. It was quantified by comparing the integral intensity of the PEG methylene protons (-O-CH_2_-CH_2_-, delta = 3.6 ppm)—or, for the poly(glutamic acid) derivative, the CH_3_- group signal (delta = 3.4 ppm)—against the integral intensity of the peak assigned to the methylene protons of the benzyl protecting group (-COOCH_2_C_6_H_5_, delta = 5.0–5.1 ppm). This analytical approach confirmed the successful preparation of N_3_-PEG_114_-*b-*pBLA_62_ (Figure S1) and mPEG_114_-*b*-pBLG_55_ (Figure S2) block copolymers.

In the subsequent step, the functionalization of the synthesized polymers was achieved via aminolysis using diethylenetriamine (**3**, **6**), spermine (**2**), or spermidine (**4**, **7**) (Figures S3–S6). The resulting cationic derivatives were purified by comprehensive dialysis against 0.01 M HCl utilizing a 6 kDa MWCO membrane, followed by lyophilization and storage under a protective argon atmosphere.

### 3.2. Cytotoxicity of Block Copolymers

The cytotoxicity of all synthesized polymers toward human embryonic kidney (HEK293) and neuroblastoma (SH-SY5Y) cells was evaluated using the WST-1 assay. Table 1 summarizes the cytotoxicity profiles of the block copolymers against both cell cultures. The individual dose–response curves for the compounds listed in Table 1 are provided in Supplementary Figures S7–S10, with the exception of the selected hit compound and its modified analog, which are displayed below in Figure 3.

Cytotoxicity profiling revealed substantial differences among the investigated polymers. Among the tested compounds, N_3_-PEG_114_-pAsp(DET)_62_ exhibited the lowest toxicity in both HEK293 cells (IC_50_ = 49.7 ± 14.9 μM) and SH-SY5Y cells (IC_50_ = 48.2 ± 11.3 μM).

However, the influence of the polymer backbone depended on the nature of the cationic substituent. For the spermidine-functionalized polymers, the polyaspartamide derivative exhibited lower IC50 values than the corresponding polyglutamide derivative in both HEK 293 and SH-SY5Y cells. Differences in susceptibility between the two cell lines were also polymer-dependent. SH-SY5Y cells showed lower IC50 values for several derivatives, whereas the spermidine-functionalized polyaspartamide exhibited comparable IC50 values in the two cell lines. Thus, neither the influence of backbone composition nor differences in cell-line sensitivity can be generalized across all investigated polymer structures.

### 3.3. Transfection Efficiency of Block Copolymers

Polyplexes for transfection were prepared by simple mixing of the block copolymer with either pTurboGFP-C or pTurboRFP-C plasmids. The N/P ratio, defined as the molar ratio of primary amino groups in the cationic block copolymers to phosphate groups in the plasmid DNA, was varied during preparation. The hydrodynamic diameter of the polyplexes, as determined by DLS, ranged from 250 to 300 nm. At N/P ratios of 5 and above, the polyplex zeta potential was approximately +30 mV, which is in good agreement with previously reported data [26]. The polyplexes were incubated with the cells for 24 h, followed by the evaluation of transfection efficiency using various methods.

**Figure 1:**
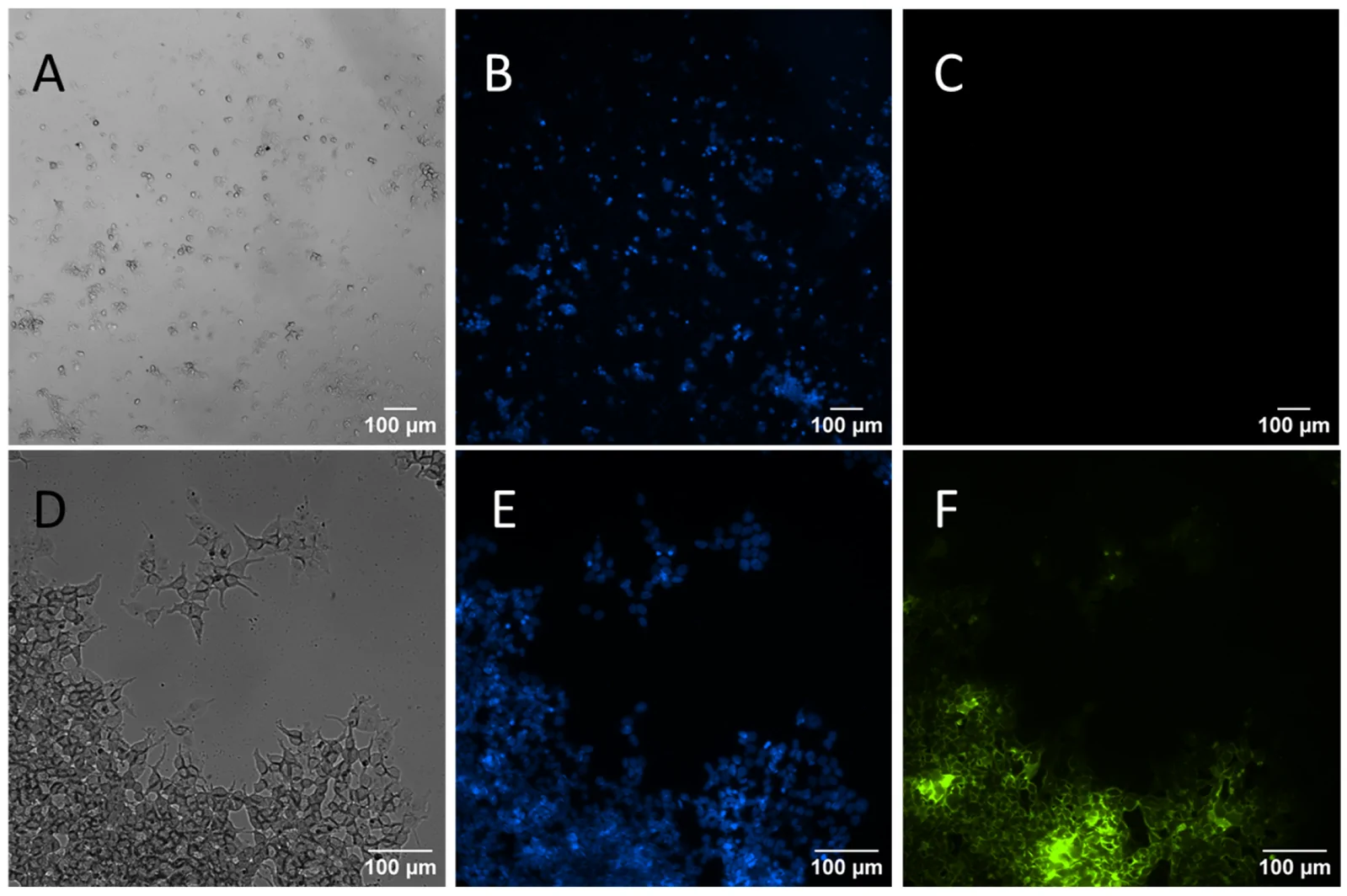
*In vitro* transfection efficiency of polyplexes in HEK293 cells. Fluorescence and brightfield microscopy images of HEK293 cells. (**A**–**C**) Untransfected control cells: (**A**) brightfield channel, (**B**) cell nuclei stained with Hoechst dye (λ_ex_ 350 nm/λ_em_ 460 nm filter set), and (**C**) GFP channel (λ_ex_ 492 nm/λ_em_ 516 nm filter set). (**D**–**F**) Cells transfected with N_3_-PEG_114_-pAsp(DET)_62_/pTurboGFP-C polyplexes: (**D**) brightfield channel, (**E**) cell nuclei stained with Hoechst dye, and (**F**) GFP channel demonstrating TurboGFP expression. Scale bar represents 100 μm.

Qualitatively, transfection efficiency was assessed via fluorescence microscopy. Cell nuclei were counterstained with Hoechst dye, with fluorescence monitored at λ_ex_ 350 nm/λ_em_ 460 nm. As shown in Figure 1, no signal was detected for untransfected HEK293 cells when using the λ_ex_ 492 nm/λ_em_ 516 nm filter set. In contrast, cells transfected with N_3_-PEG_114_-pAsp(DET)_62_/pTurboGFP-C polyplexes exhibited intense fluorescence in the same region as the stained cell nuclei (λ_ex_ 350 nm/λ_em_ 460 nm filter set), indicating successful reporter expression.

**Figure 2:**
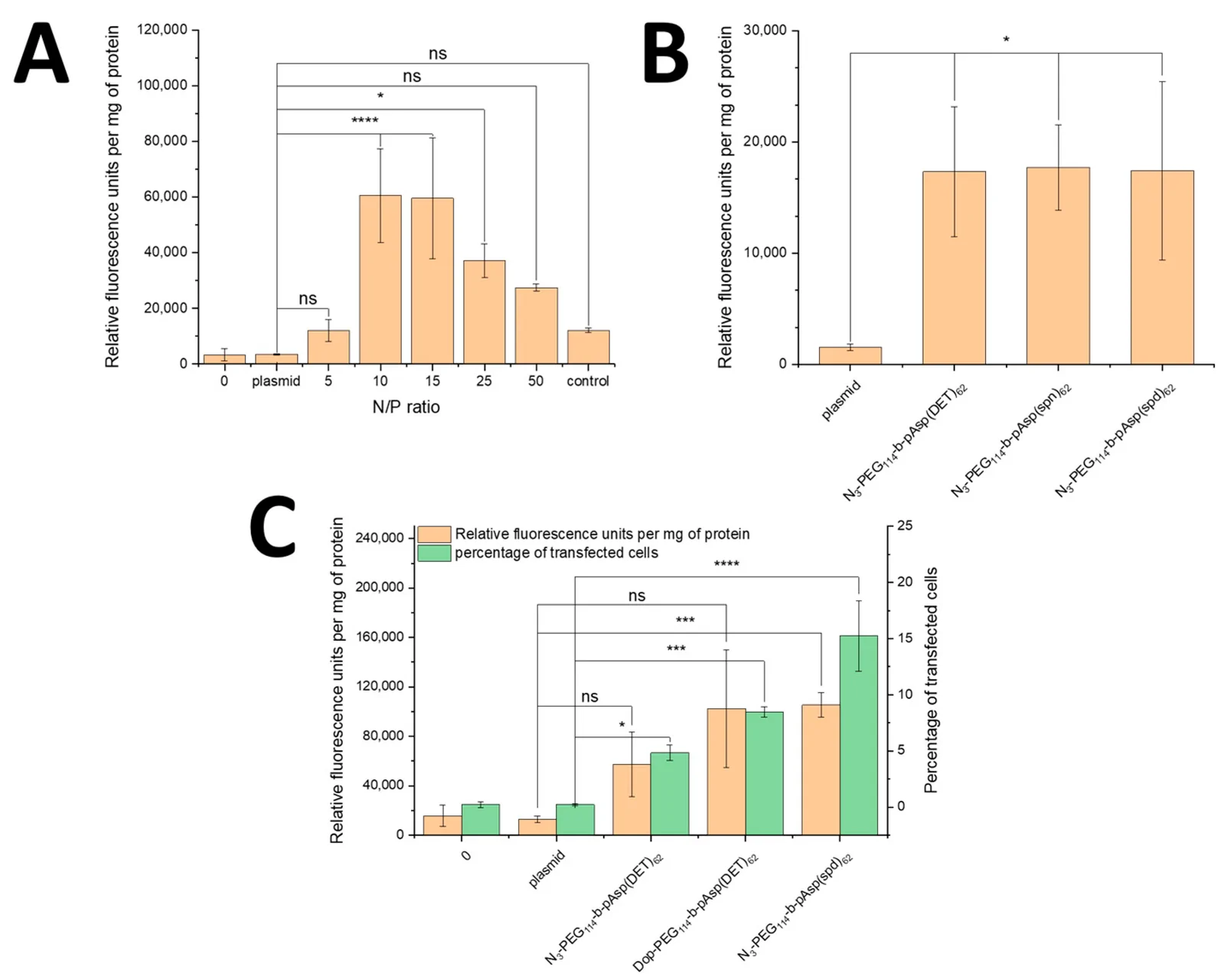
(**A**) Quantitative evaluation of *in vitro* transfection efficiency of N_3_-PEG_114_-b-pAsp(DET)_62_/pDNA polyplexes at various N/P ratios (5, 10, 15, 25, and 50) in HEK293 cells, expressed as relative fluorescence units (RFU) per mg of total protein. “0” represents untreated cells; “plasmid” indicates cells incubated with naked pTurboGFP-C pDNA; PEG_114_-b-PLL_50_/pDNA polyplexes serve as a positive control. Cells were incubated with polyplexes (4.5 μg/mL pDNA) for 24 h, followed by a 24 h post-incubation in fresh complete medium. Fluorescence intensity of cell lysates was measured fluorometrically and normalized to total protein content. Data are presented as mean ± SD (*n* = 3). (**B**) Comparative in vitro transfection efficiency of various polyplex formulations in SH-SY5Y cells. Evaluation of transfection efficiency for N_3_-PEG_114_-b-pAsp(DET)_62_, N_3_-PEG_114_-b-pAsp(spn)_62_, and N_3_-PEG_114_-b-pAsp(spd)_62_ polyplexes carrying pTurboRFP-C pDNA. Transfection efficiency is expressed as relative fluorescence units (RFU) per mg of total protein. “Plasmid” indicates control cells incubated with naked pTurboRFP-C pDNA. Cells were incubated with polyplexes (4.5 μg/mL pDNA) for 24 h, followed by a 24 h post-incubation in fresh complete medium. Fluorescence intensity of cell lysates was measured fluorometrically and normalized to total protein content. Data are presented as mean ± SD (*n* = 3). (**C**) Evaluation of transfection efficiency by total fluorescence and percentage of transfected cells. Comparative transfection profiles of various functionalized polyplexes in SH-SY5Y cells. The left Y-axis (orange bars) represents relative fluorescence units (RFU) per mg of total protein. The right Y-axis (green bars) represents the percentage of transfected cells measured by flow cytometry. “0” indicates untreated cells; “plasmid” indicates cells incubated with naked pDNA; “Dop” denotes dopamine-functionalized block copolymer. Cells were incubated with polyplexes (4.5 μg/mL pDNA) for 24 h, followed by a 24 h post-incubation in fresh complete medium. Data are presented as mean ± SD (*n* = 3). All experiments were performed in triplicate. Asterisks indicate statistically significant differences compared with the naked plasmid control (one-way ANOVA followed by Dunnett’s post hoc test). Statistical significance: * *p* < 0.05; *** *p* < 0.001; **** *p* < 0.0001; ns, not significant (*p* ≥ 0.05).

To quantitatively evaluate transfection efficiency, an assay measuring the specific fluorescence of cell lysates was employed (Figure 2A). The total protein concentrations of the treated HEK293 cells were determined using the BCA assay and are summarized in Supplementary Table S1.

When assessing the transfection performance of the plasmid containing the TurboGFP gene, bare plasmid DNA was found to transfect HEK293 cells with negligible efficiency. However, the encapsulation of the plasmid into polyplexes based on the N_3_-PEG_114_-b-pAsp(DET)_62_ block copolymer substantially enhanced delivery outcomes. A comparison of transfection profiles at various N/P ratios revealed that gene delivery efficiency at N/P ratios of 10 and 15 was significantly higher than that observed at N/P = 5. For all studied N/P ratios, the concentration of N_3_-PEG_114_-b-pAsp(DET)_62_ block copolymer within the well was substantially lower than the IC_50_ value.

Furthermore, evaluating the delivery efficiency of pTurboGFP-C/N_3_-PEG_114_-b-pAsp(DET)_62_ polyplexes into HEK293 cells at N/P ratios of 25 and 50 demonstrated superior performance at N/P = 25. Notably, the synthesized N_3_-PEG_114_-b-pAsp(DET)_62_ block copolymer outperformed a commercially available PEG_114_-pLL_50_ reference polymer, which exhibited lower transfection efficiency under identical conditions.

Transfection of SH-SY5Y neuroblastoma cells was initially performed using bare plasmid DNA and various polyplex formulations at a fixed N/P ratio of 15 (Figure 2B). Subsequent comparative experiments were conducted using an N/P ratio of 15, which afforded high reporter gene expression at a reduced polymer concentration compared to higher N/P ratios. When the bare pTurboRFP-C plasmid was employed, the delivery efficiency into SH-SY5Y cells was negligible. In contrast, the utilization of polyplexes significantly enhanced transfection efficiency, with all tested polymers demonstrating comparable levels of protein expression. For the studied N/P ratio of 15, the concentration of block-copolymer N_3_-PEG_114_-b-pAsp(DET)_62_ within the well remained substantially lower than the IC_50_, while those of N_3_-PEG_114_-b-pAsp(spn)_62_ and N_3_-PEG_114_-b-pAsp(spd)_62_ reached the IC_50_ threshold. Nevertheless, all polymers exhibited similar transfection performance. Total protein levels measured by the BCA method are provided in Supplementary Table S2.

The lead polymer **3** was subsequently subjected to dopamine functionalization using a one-pot coupling procedure. Following the reaction, the product **8** was extensively purified by prolonged dialysis using a 6 kDa molecular weight cut-off (MWCO) membrane with repeated replacement of the dialysis medium to remove unreacted dopamine and other low-molecular-weight reaction components.

**Figure 3:**
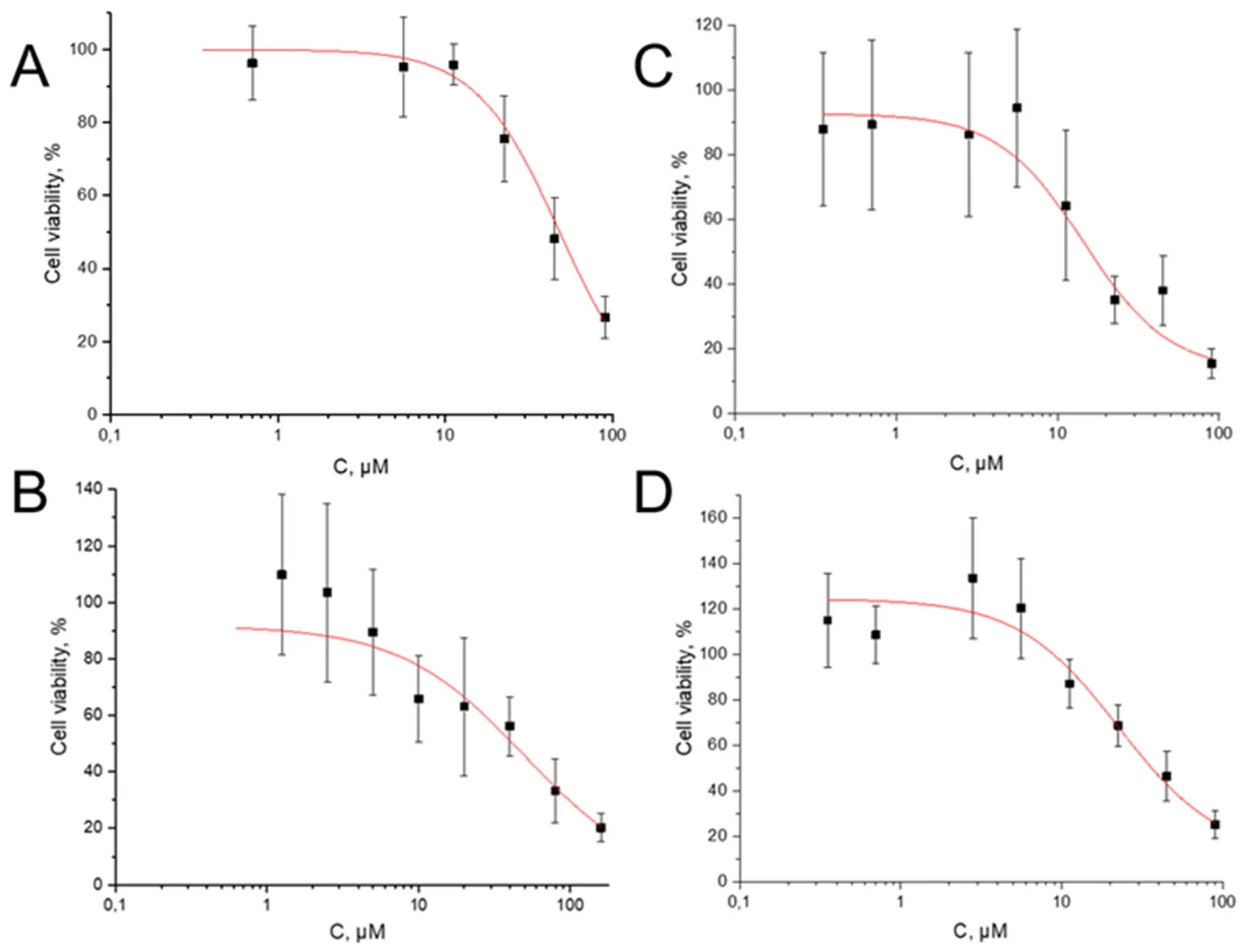
Dose–response curves for block copolymers against human neuroblastoma and embryonic kidney cells. Cell viability was assessed via the WST-1 assay following 24 h of incubation. (**A**,**B**) Cytotoxicity profiles of the parent N_3_-PEG_114_-b-pAsp(DET)_62_ polymer against (**A**) SH-SY5Y and (**B**) HEK293 cell lines. (**C**,**D**) Cytotoxicity profiles of the dopamine-functionalized Dop-PEG_114_-b-pAsp(DET)_62_ against (**C**) SH-SY5Y and (**D**) HEK293 cell lines. Values shown are mean ± SD (*n* = 3).

The cytotoxicity of the dopamine-functionalized polymer was evaluated using the WST-1 assay. Figure 3 illustrates the comparative concentration-dependent cell viability curves for both the parent and functionalized polymers. Dopamine modification of N_3_-PEG_114_-b-pAsp(DET)_62_ was associated with a decrease in IC_50_ from 49.7 ± 8.4 to 22.7 ± 12.5 µM in HEK 293 cells and from 48.8 ± 11.3 to 14.5 ± 5.0 µM in SH-SY5Y cells. These changes correspond to approximately 2.2-fold and 3.3-fold lower IC_50_ values, respectively, indicating increased cytotoxic potency of the modified material under the conditions studied.

Subsequently, the transfection efficiency in SH-SY5Y neuroblastoma cells was comprehensively evaluated using two distinct quantitative methods: specific fluorescence analysis of cell lysates and flow cytometry (Figure 2C). Functionalization of the polymer with a dopamine moiety resulted in a 1.78-fold increase in protein-normalized fluorescence and a 1.75-fold increase in the percentage of GFP-positive cells, respectively. For these experiments, cells were transfected at a constant N/P ratio of 15 using either bare plasmid DNA, non-targeted polyplexes (pTurboGFP-C/N_3_-PEG_114_-b-pAsp(DET)_62_ or pTurboGFP-C/N_3_-PEG_114_-b-pAsp(spd)_62_), or dopamine-receptor-targeted polyplexes (pTurboGFP-C/Dop-PEG_114_-b-pAsp(DET)_62_). The concentrations of both DET-based block copolymers were well below the IC_50_, while that of polymer N_3_-PEG_114_-b-pAsp(spd)_62_ remained at the IC_50_ threshold.

Flow cytometry analysis of transfection efficiency revealed that bare plasmid DNA exhibited almost no intracellular uptake, whereas the application of polymers as delivery vectors substantially enhanced reporter gene expression (corresponding raw cytometric profiles are provided in Supplementary Figures S11 and S12). The N_3_-PEG_114_-b-pAsp(spd)_62_ polymer demonstrated the highest performance, yielding a transfection rate of 15.2 ± 3.1%. Furthermore, conjugation of the polymer with dopamine was found to increase transfection efficiency.

An identical trend was observed when evaluating transfection efficiency via fluorescence analysis of cell lysates normalized to total protein content. Protein quantification via the BCA assay is detailed in Supplementary Table S3. The only notable discrepancy was a reduced difference in signal intensities between the polyplexes prepared with N_3_-PEG_114_-b-pAsp(DET)_62_ and those with N_3_-PEG_114_-b-pAsp(spd)_62_.

## 4. Discussion

The primary objective of this study was to develop a versatile polymeric platform for nucleic acid delivery. To achieve this, a series of block copolymers was synthesized based on polyaspartamide or polyglutamide backbones, which have been investigated as potentially biodegradable components of non-viral delivery systems, and polyethylene glycol (PEG) segments, incorporated to improve the physicochemical properties and potential biocompatibility of the resulting carriers.

Cytotoxicity profiling of the synthesized block copolymers revealed that the N_3_-PEG_114_-pAsp(DET)_62_ formulation possessed the highest biocompatibility. Its low cytotoxicity is hypothesized to stem from differences in the cationic side-chain architecture and effective charge characteristics. Additionally, this trend may also be related to the oxidative metabolism of polyamines. However, these properties were not directly quantified in the present study.

The relative cytotoxicity of the polyaspartamide- and polyglutamide-based derivatives depended on the attached polyamine. Previously reported differences in degradation behavior may contribute to these observations; however, degradation kinetics were not evaluated in the present study [13]. A contribution of the different PEG terminal groups cannot be excluded. An alternative explanation involves the catabolism of spermine and spermidine into toxic aldehydes and hydrogen peroxide, which induce severe oxidative stress; notably, spermine undergoes more intense oxidation [30,31].

Differences in cytotoxicity between HEK 293 and SH-SY5Y cells were dependent on the polymer structure. The heightened cytotoxicity of a number of polymers toward SH-SY5Y cells is likely due to the intrinsic susceptibility of this neuroblastoma line to cytotoxic agents [32], whereas the sharp increase in the toxicity of the spermidine-containing polymer highlights the extreme sensitivity of neuronal cells to oxidative stress. Furthermore, modification of N_3_-PEG_114_-b-pAsp(DET)_62_ with dopamine was found to elevate cytotoxicity against both cell lines. Specifically, in the non-neuronal HEK293 cell line, cytotoxicity increased 2.2-fold, which is likely associated with non-specific interactions between the dopamine residue and cell membrane components. Conversely, in the SH-SY5Y cell line, a 3.3-fold increase in cytotoxicity was recorded; this may reflect altered interactions of the modified material with cellular components.

Polyplex formation substantially enhanced the functional intracellular delivery of pDNA, as evidenced by increased reporter gene expression. An N/P ratio of 15 was selected for subsequent experiments, as it provided substantial reporter gene expression at a lower polymer concentration than formulations prepared at higher N/P ratios. Below this value, reporter gene expression efficiency decreases substantially, presumably due to an insufficient positive charge on the polymer. Conversely, a high N/P ratio also leads to a decline in delivery efficiency; this phenomenon may occur because reporter protein expression reaches saturation while the concomitant cytotoxicity continues to escalate.

In the case of SH-SY5Y neuroblastoma cells, the enhanced delivery efficiency achieved with dopamine-modified polyplexes compared to their unmodified counterparts is consistent with the expression of dopamine receptors on the surface of these cells [33]. However, the involvement of dopamine receptors in the observed enhancement was not directly evaluated. Finally, the discrepancy in transfection performance between diethylenetriamine- and spermidine-based formulations may be related to differences in cationic side-chain composition and the resulting physicochemical properties of the polyplexes.

The material obtained following the dopamine-functionalization procedure exhibited increased reporter gene expression in SH-SY5Y cells compared with the corresponding non-functionalized polymer. Importantly, this effect was independently reproduced using two complementary readouts: fluorescence measurements in cell lysates and flow-cytometric analysis. The agreement between the two analytical approaches supports a consistent functional effect associated with dopamine functionalization.

The present study has several limitations. First, the degrees of substitution of the aminolyzed derivatives could not be independently quantified for all polymers, and the molecular masses used for concentration calculations were based on nominal structural compositions. Second, the final material obtained following dopamine conjugation was not directly characterized by spectroscopic or other quantitative analytical methods, and the degree of dopamine substitution remains unknown. Although extensive dialysis was performed, residual dopamine and copper were not quantified, and their complete removal cannot be confirmed. Third, the physicochemical properties of the resulting polyplexes, including their effective surface charge, were not directly determined. Therefore, the observed differences in cytotoxicity and reporter gene expression cannot be conclusively attributed to specific charge-dependent or degradation-related mechanisms. Finally, receptor binding, receptor-dependent internalization, and neuronal selectivity were not investigated. Consequently, the enhanced reporter gene expression observed after dopamine modification should be regarded as a functional association rather than evidence of confirmed receptor-mediated targeting. Further chemical characterization and mechanistic investigations will be required to establish the underlying delivery mechanisms.

## 5. Conclusions

In conclusion, a series of cationic block copolymers based on polyaspartamide or polyglutamide and polyethylene glycol (PEG) was successfully synthesized via a two-step procedure and evaluated. This study demonstrates that the architectural and chemical features of the polymer backbones exert a profound influence on both their biocompatibility and nucleic acid delivery performance.

The investigated polymers exhibited distinct cytotoxicity profiles. However, the relative cytotoxicity of the polyaspartamide- and polyglutamide-based polymers depended on the nature of the cationic side chains, preventing a general conclusion regarding the superiority of either backbone. Cytotoxicity profiling identified N_3_-PEG_114_-b-pAsp(DET)_62_ as the most biocompatible carrier among the synthesized series.

Polyplex-mediated delivery enhanced reporter gene expression compared with naked plasmid DNA under the investigated conditions. Moreover, the material obtained following dopamine functionalization exhibited increased reporter gene expression in SH-SY5Y cells compared with the corresponding unmodified carrier. Flow cytometry and specific fluorescence assays confirmed that the engineered polyplexes, particularly the spermidine- and dopamine-functionalized derivatives, significantly outperform bare plasmid DNA and show superior or comparable transfection outcomes to the commercially available PEG_114_-pLL_50_ reference polymer.

Taken together, these findings establish the developed block copolymers as a highly adaptable and efficient polymeric platform for targeted gene delivery applications. Nevertheless, further chemical characterization of the dopamine-containing material and mechanistic investigation of cellular interactions are required before receptor-mediated targeting or selective neuronal delivery can be established.

## Figures and Tables

**Table 1:** *In vitro* cytotoxicity of block copolymers to HEK293 and SH-SY5Y cell lines. Cells were exposed to block copolymers at various concentrations for 24 h. Twenty-four hours later, cell viability was assessed using the WST-1 reagent assay. Values shown are the mean ± SD of *n* = 3 independent measurements.

Polymer Composition	IC_50_ μM, HEK293	IC_50_ μM, SH-SY5Y
N_3_-PEG_114_-b-pAsp(DET)_62_	49.7 ± 14.9	48.2 ± 11.3
N_3_-PEG_114_-b-pAsp(spn)_62_	10.0 ± 6.7	0.6 ± 0.1
N_3_-PEG_114_-b-pAsp(spd)_62_	0.8 ± 0.1	0.8 ± 0.2
mPEG_114_-b-pGlu(spd)_55_	4.0 ± 4.9	2.6 ± 0.3
mPEG_114_-b-pGlu(DET)_55_	7.1 ± 3.2	n.d. ^1^

^1^ n.d., not determined. The IC50 value for polymer mPEG_114_-b-pGlu(DET)_55_ in SH-SY5Y cells was not determined; this polymer was not selected for subsequent functionalization and was not used for the downstream comparative transfection studies.

## Data Availability

The original contributions presented in this study are included in the article and Supplementary Materials. Further inquiries can be directed to the corresponding authors.

## Supplementary Materials

The following supporting information can be downloaded at https://www.mdpi.com/article/10.3390/polym18212671/s1, Figure S1: The ^1^H-NMR spectra of N_3_-PEG_114_-b-pBLA_62_; Figure S2: The ^1^H-NMR spectra of mPEG_114_-b-pBLG_55_; Figure S3: The ^1^H-NMR spectra of N_3_-PEG_114_-b-pAsp(DET)_62_; Figure S4: The ^1^H-NMR spectra of mPEG_114_-b-pGlu(spd)_55_; Figure S5: The ^1^H-NMR spectra of N_3_-PEG_114_-b-pAsp(spd)_62_; Figure S6: The ^1^H-NMR spectra of N_3_-PEG_114_-b-pAsp(spn)_62_; Figure S7: Dose–response curves for the N_3_-PEG_114_-b-pAsp(spn)_62_; Figure S8: Dose–response curves for the N_3_-PEG_114_-b-pAsp(spd)_62_; Figure S9: Dose–response curves for the mPEG_114_-b-pGlu(spd)_55_; Figure S10: Dose–response curves for the mPEG_114_-b-pGlu(spd)_55_; Table S1: The protein concentration (mg/mL) after HEK 293 cells transfection using N_3_-PEG_114_-b-pAsp(DET)_62_/pDNA; Table S2: The protein concentration (mg/mL) after SH-SY5Y cell transfection using N_3_-PEG_114_-b-pAsp(DET)_62_, N_3_-PEG_114_-b-pAsp(spn)_62_, and N_3_-PEG_114_-b-pAsp(spd)_62_ polyplexes carrying pTurboRFP-C pDNA; Table S3: The protein concentration (mg/mL) after SH-SY5Y cell transfection using either bare plasmid DNA, non-targeted polyplexes (pTurboGFP-C/N_3_-PEG_114_-b-pAsp(DET)_62_ or pTurboGFP-C/N_3_-PEG_114_-b-pAsp(spd)_62_), or dopamine-receptor-targeted polyplexes (pTurboGFP-C/Dop-PEG_114_-b-pAsp(DET)_62_); Figures S11 and S12: Characterization of SH-SY5Y cell transfection via flow cytometry.

## Abbreviations

The following abbreviations are used in this manuscript:

BCA	bicinchoninic acid
BLA-NCA	β-benzyl-L-aspartate N-carboxyanhydride
BLG-NCA	γ-benzyl-L-glutamate N-carboxyanhydride
DET	diethylenetriamine
DMEM/F12	Dulbecco’s modified Eagle medium/Nutrient Mixture F-12
DMF	N,N-dimethylformamide
DMSO	dimethyl sulfoxide
DPBS	Dulbecco’s phosphate-buffered saline
FBS	fetal bovine serum
GFP	green fluorescent protein
IC_50_	half-maximal inhibitory concentration
MWCO	molecular weight cut-off
N/P ratio	nitrogen-to-phosphate ratio
P/S	penicillin/streptomycin
pAsp	polyaspartamide-based block
pBLA	poly(β-benzyl-L-aspartate)
pBLG	poly(γ-benzyl-L-glutamate)
pDNA	plasmid DNA
PEG	poly(ethylene glycol)
pGlu	polyglutamide-based block
PLL	poly-L-lysine
RFU	relative fluorescence units
RFP	red fluorescent protein
SFM	serum-free medium
spd	spermidine
spn	spermine
