# Supplementary material for: Influence of Polymer Backbone Architecture and Amino Group Density on the Cytotoxicity and Transfection Performance of Cationic Block Copolymer Polyplexes
Source: Polymers (Basel). 2026 Oct 28;18(21):2671. doi: 10.3390/polym18212671 (PMC13765683; doi:10.3390/polym18212671)
Supplement: Supplementary file 1 [file polymers-18-02671-s001.zip › polymers-4629698-supplementary.pdf]

Influence of Polymer Backbone Architecture and Amino Group Density on the Cytotoxicity and Transfection Performance of Cationic Block Copolymer Polyplexes

Nikita A. Kozyrev, Alexey V. Doroshchenko, Anna D. Lapanik, Natalia L. Klyachko, Anton V. Lopukhov

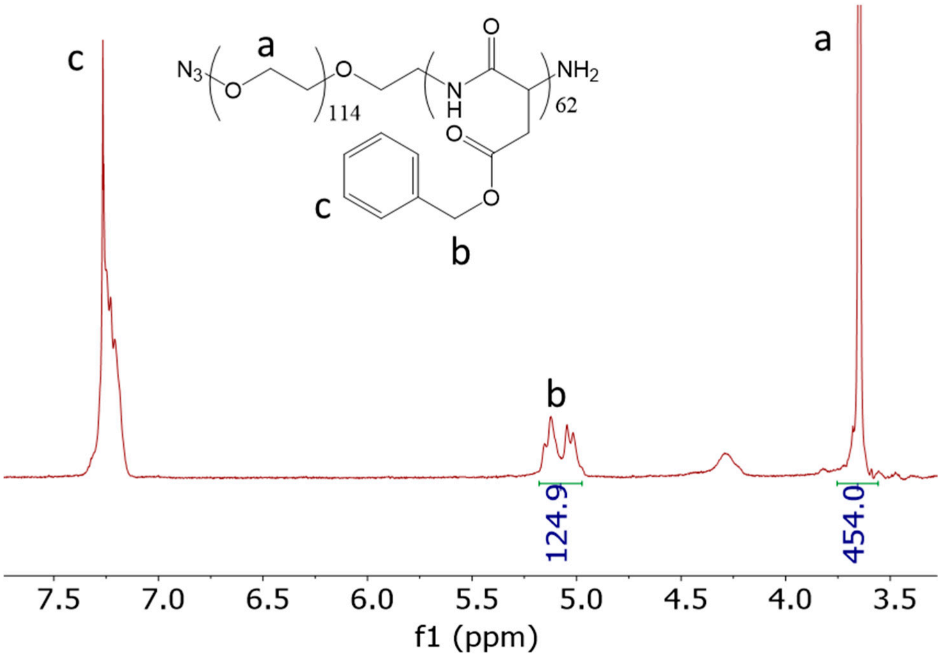

Figure S1. The ^{1}H-NMR spectra of N_3-PEG_{114}-b-$pBLA_{62}$ ($CDCl_3$, 400 MHz, 25 °C).

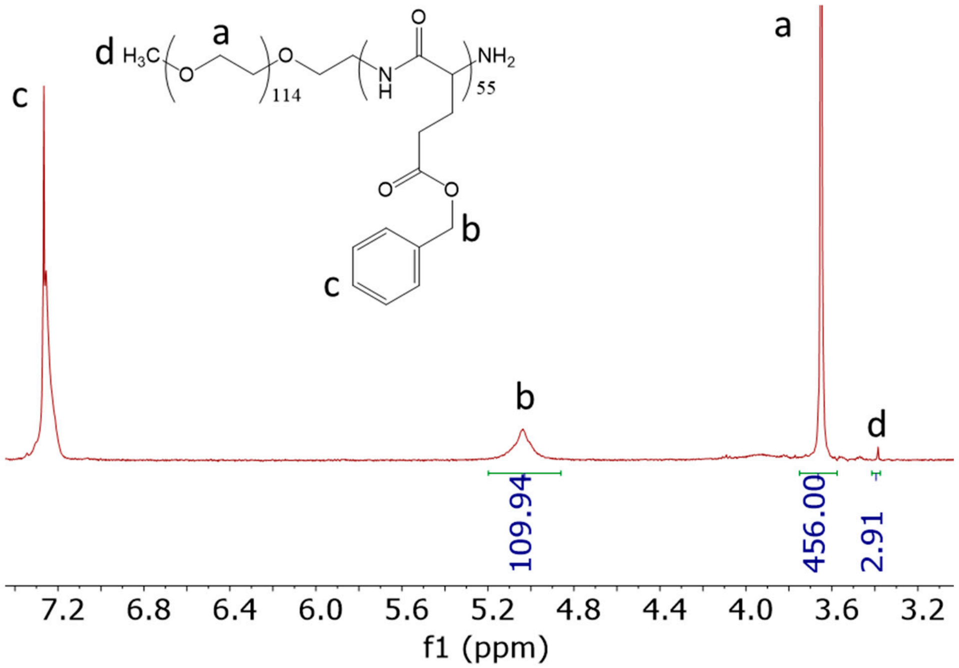

Figure S2. The ^{1}H-NMR spectra of mPEG$_{114}$-b-pBLG$_{55}$ ($CDCl_3$, 400 MHz, 25 °C)

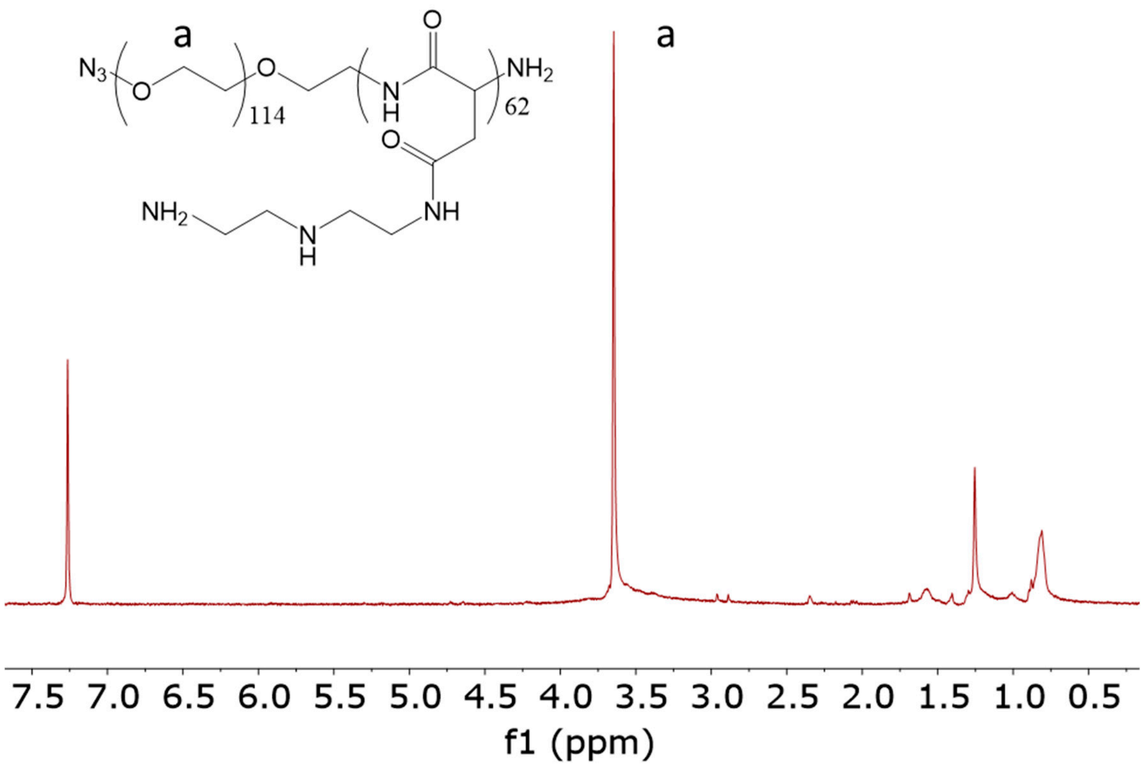

Figure S3. The ^{1}H-NMR spectra of N$_3$-PEG$_{114}$-b-pAsp(DET)$_{62}$ ($CDCl_3$, 400 MHz, 25 °C)

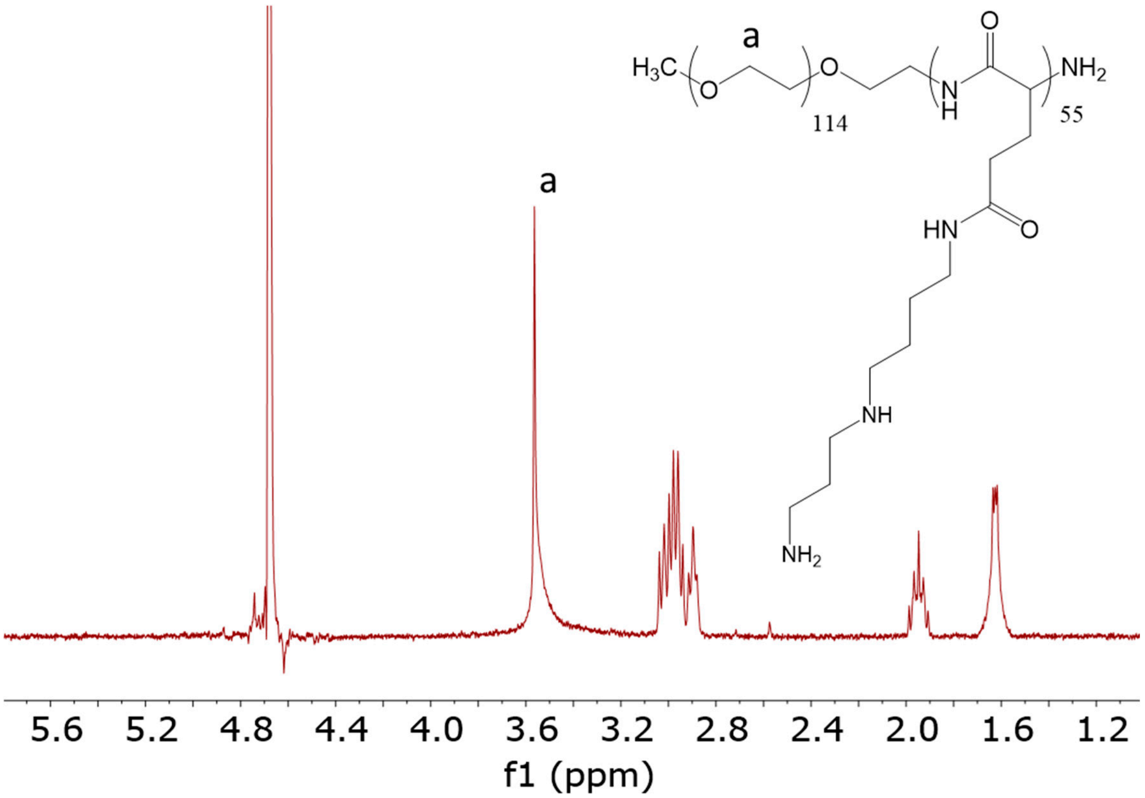

Figure S4. The ^{1}H-NMR spectra of mPEG$_{114}$-b-pGlu(spd)$_{55}$ (D_2O, 400 MHz, 25 °C).

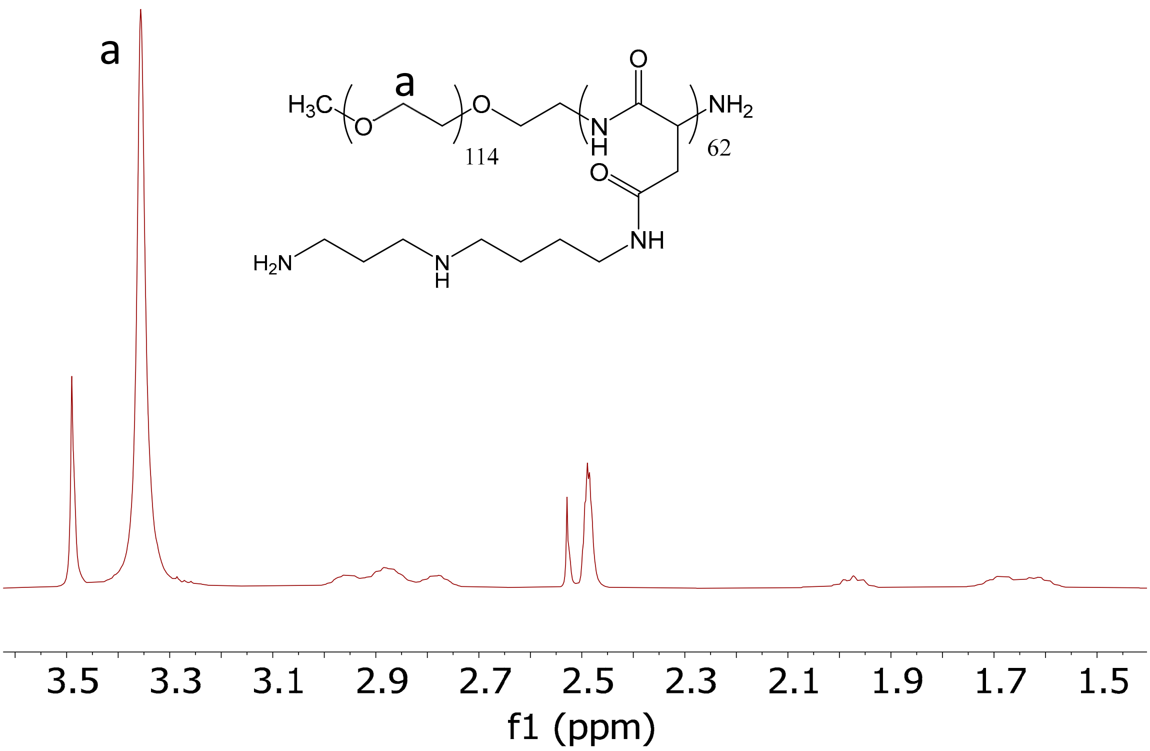

Figure S5. The ^{1}H-NMR spectra of N_3-PEG_{114}-b-pAsp(spd)$_{62}$ (D_2O, 400 MHz, 25 °C).

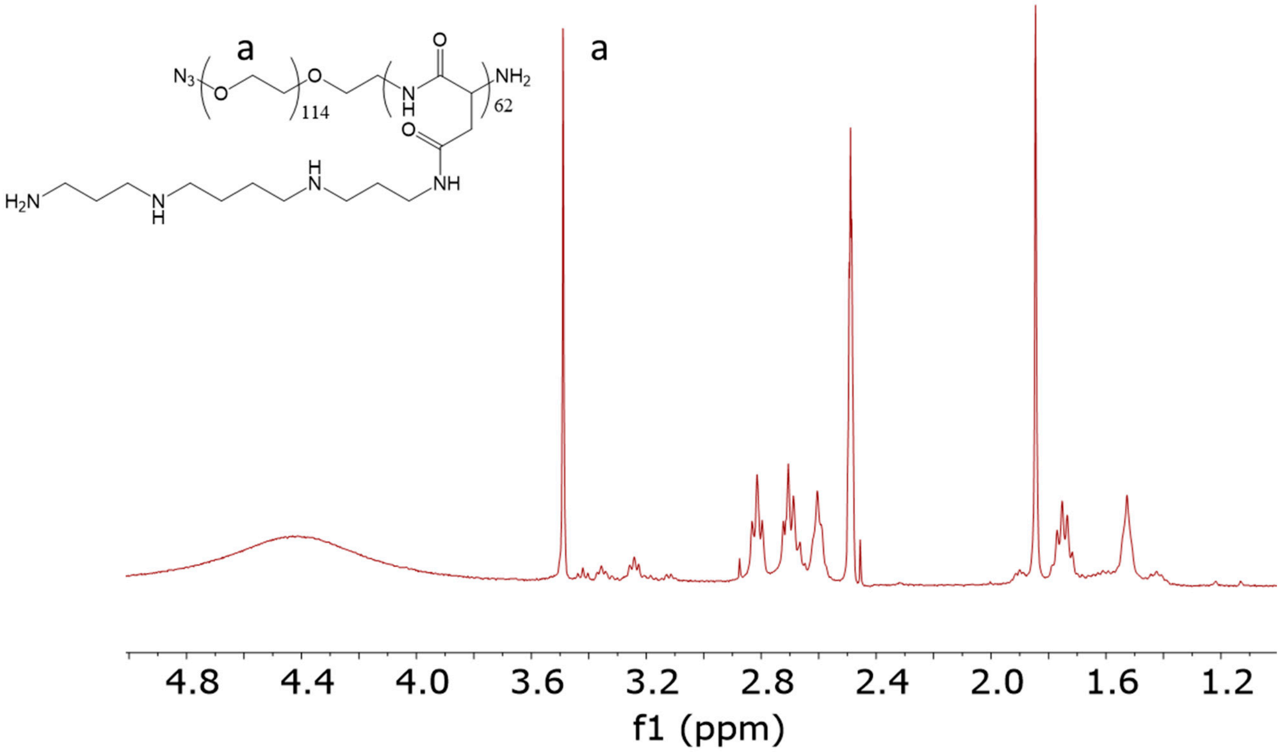

Figure S6. The ^{1}H-NMR spectra of N_3-PEG_{114}-b-pAsp(spn)$_{62}$ (DMSO-d_6, 400 MHz, 25 °C).

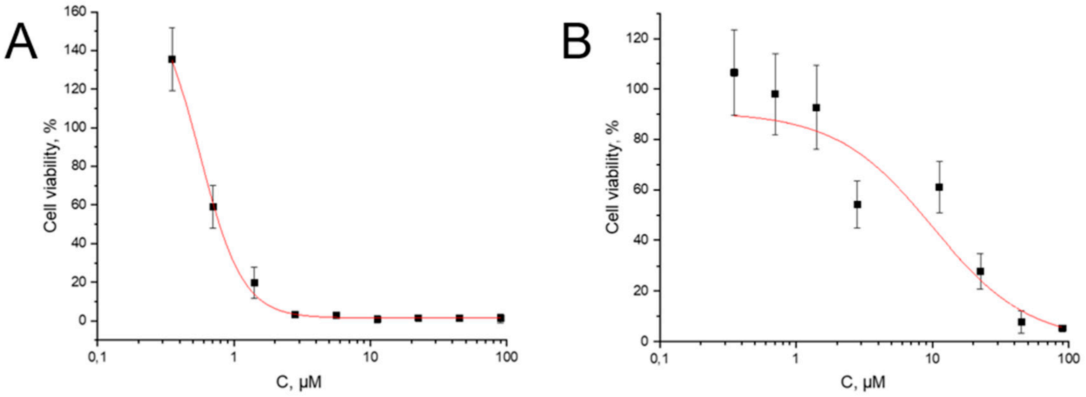

Figure S7. *In vitro* cytotoxicity of N_3-PEG_{114}-b-pAsp(spn)$_{62}$ block copolymer to (A) SH-SY5Y and (B) HEK293 cell lines. Cells were exposed to block copolymer at various concentrations for 24 hrs. Twenty-four hours later, cell viability was assessed by WST-1 assay. Values shown are mean ± SD (n = 3).

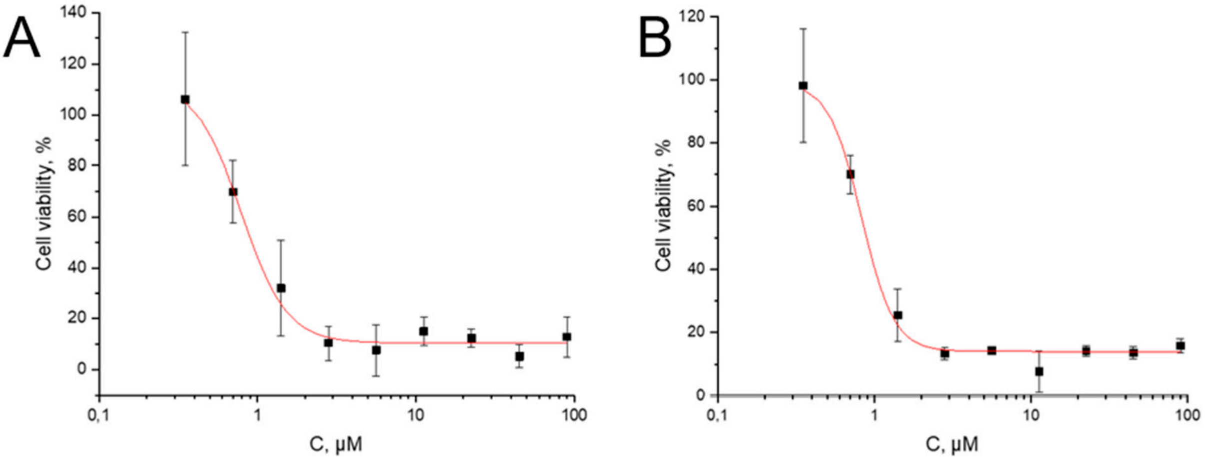

Figure S8. *In vitro* cytotoxicity of N_3-PEG_{114}-b-pAsp(spd)$_{62}$ block copolymer to (A) SH-SY5Y and (B) HEK293 cell lines. Cells were exposed to block copolymer at various concentrations for 24 hrs. Twenty-four hours later, cell viability was assessed by WST-1 assay. Values shown are mean ± SD (n = 3).

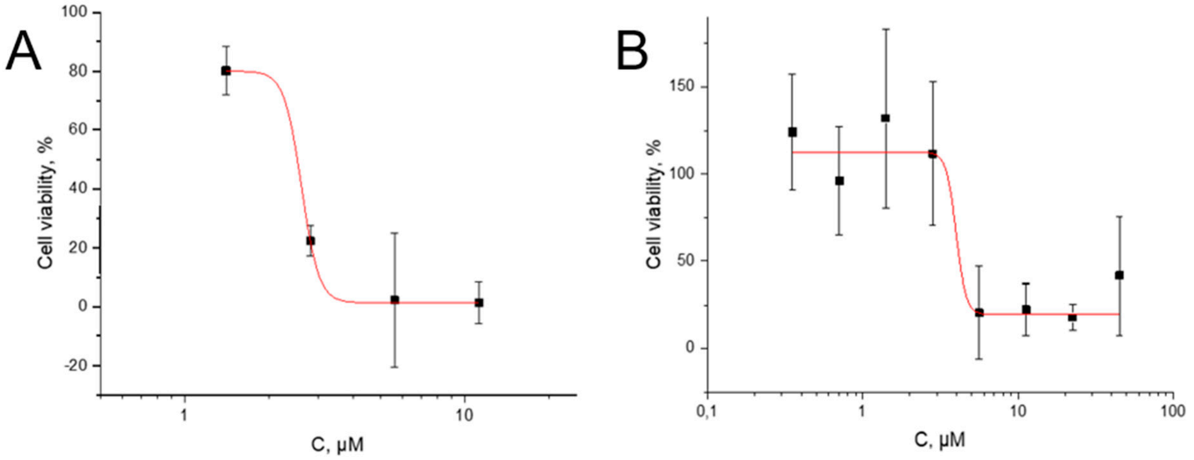

Figure S9. *In vitro* cytotoxicity of mPEG$_{114}$-b-pGlu(spd)$_{55}$ block copolymer to (A) SH-SY5Y and (B) HEK293 cell lines. Cells were exposed to block copolymer at various concentrations for 24 hrs. Twenty-four hours later, cell viability was assessed by WST-1 assay. Values shown are mean ± SD (n = 3).

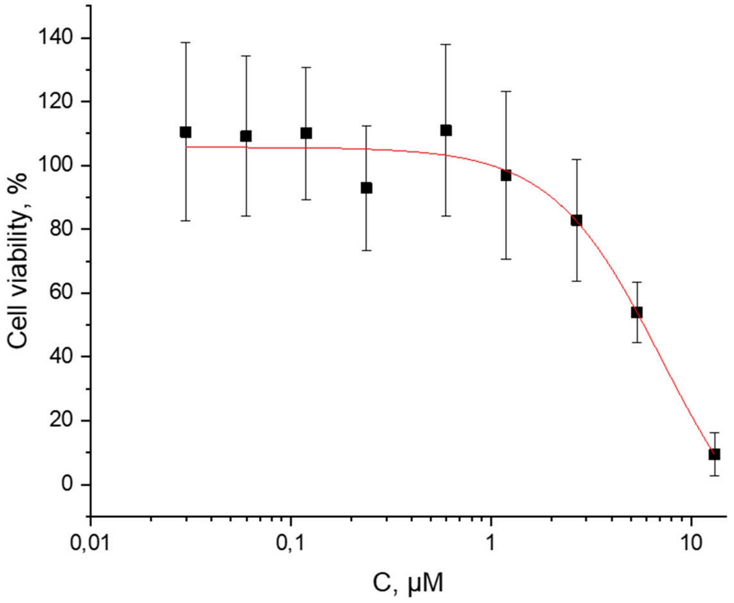

Figure S10. *In vitro* cytotoxicity of mPEG$_{114}$-b-pGlu(DET)$_{55}$ block copolymer to HEK293 cell lines. Cells were exposed to block copolymer at various concentrations for 24 hrs. Twenty-four hours later, cell viability was assessed by WST-1 assay. Values shown are mean ± SD (n = 3).

Table S1. The protein concentration (mg/ml) determined using BCA assay after treating HEK 293 cells transfected using N$_3$-PEG$_{114}$-b-pAsp(DET)$_{62}$/pDNA with lysis buffer for 2 hrs. Values are mean ± SD (n = 3).

	N$_3$-PEG$_{114}$-b-pAsp(DET)$_{62}$/pDNA N/P ratio							
	0	plasmide	5	10	15	25	50	control
protein concentration	1.92 ± 1.41	2.29 ± 0.81	1.84 ± 1.10	0.88 ± 0.55	1.50 ± 0.69	0.81 ± 0.13	0.90 ± 0.03	0.59 ± 0.XX

(mg/ml)

Table S2. The protein concentration (mg/ml) determined using BCA assay after treating SH-SY5Y cells transfected using N_3-PEG$_{114}$-b-pAsp(DET)$_{62}$, N_3-PEG$_{114}$-b-pAsp(spn)$_{62}$, and N_3-PEG$_{114}$-b-pAsp(spd)$_{62}$ polyplexes carrying pTurboRFP-C pDNA with lysis buffer for 2 hrs. Values are mean ± SD (n = 3).

	plasmide	N_3-PEG$_{114}$-b-pAsp(DET)$_{62}$	N_3-PEG$_{114}$-b-pAsp(spn)$_{62}$	N_3-PEG$_{114}$-b-pAsp(spd)$_{62}$
protein concentration (mg/ml)	0.68 ± 0.03	0.75 ± 0.12	0.46 ± 0.09	0.49 ± 0.07

Table S3. The protein concentration (mg/ml) determined using BCA assay after treating SH-SY5Y cells transfected using either bare plasmid DNA, non-targeted polyplexes (pTurboGFP-C/N_3-PEG$_{114}$-b-pAsp(DET)$_{62}$ or pTurboGFP-C/N_3-PEG$_{114}$-b-pAsp(spd)$_{62}$), or dopamine-receptor-targeted polyplexes (pTurboGFP-C/Dop-PEG$_{114}$-b-pAsp(DET)$_{62}$) with lysis buffer for 2 hrs. Values are mean ± SD (n = 3).

	0	plasmide	N_3-PEG$_{114}$-b-pAsp(DET)$_{62}$	Dop-PEG$_{114}$-b-pAsp(DET)$_{62}$	N_3-PEG$_{114}$-b-pAsp(spd)$_{62}$
protein concentration (mg/ml)	1.31 ± 0.44	1.26 ± 0.37	1.08 ± 0.43	1.27 ± 0.63	0.59 ± 0.04

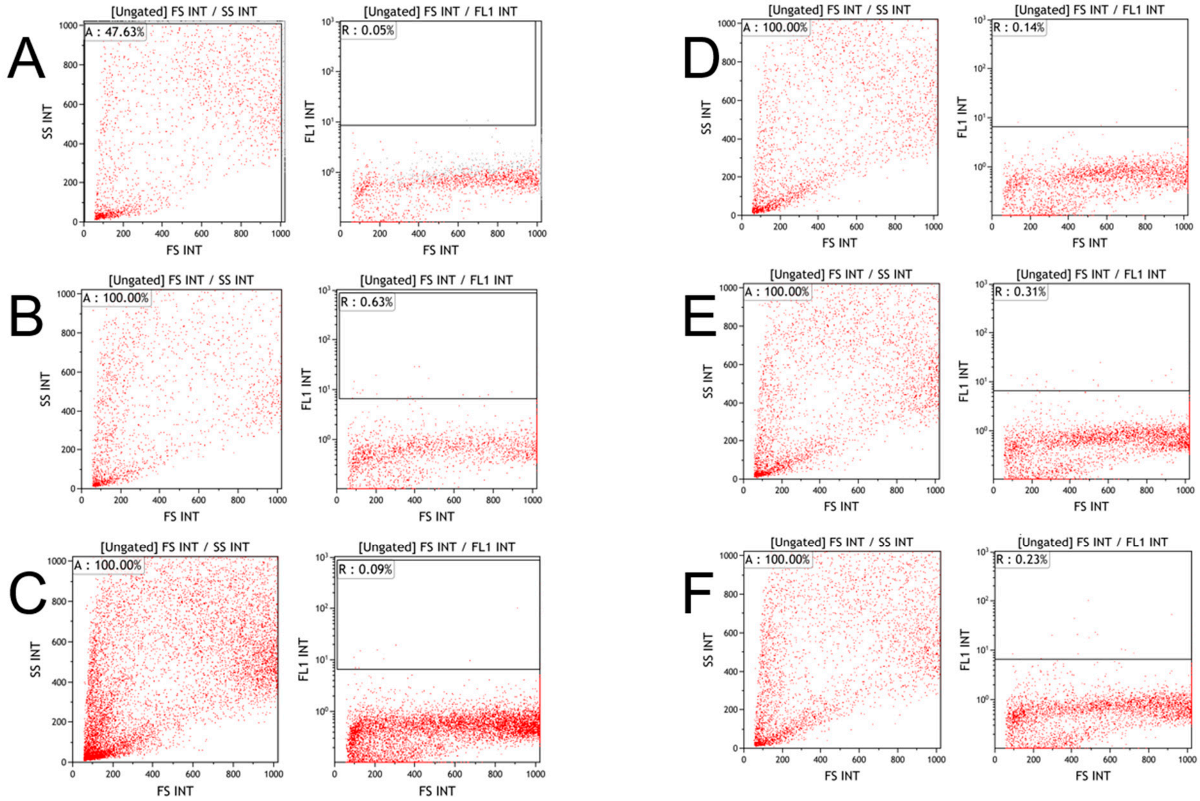

Figure S11. Characterization of SH-SY5Y cell transfection via flow cytometry. Representative dot plots display forward scatter (FS int) versus side scatter (SS int) parameters (left panels) and FS int versus FL1 fluorescence intensity (right panels) for (A-C) PBS-treated control cells and (D-F) cells transfected with naked pTurboGFP-C plasmid DNA.

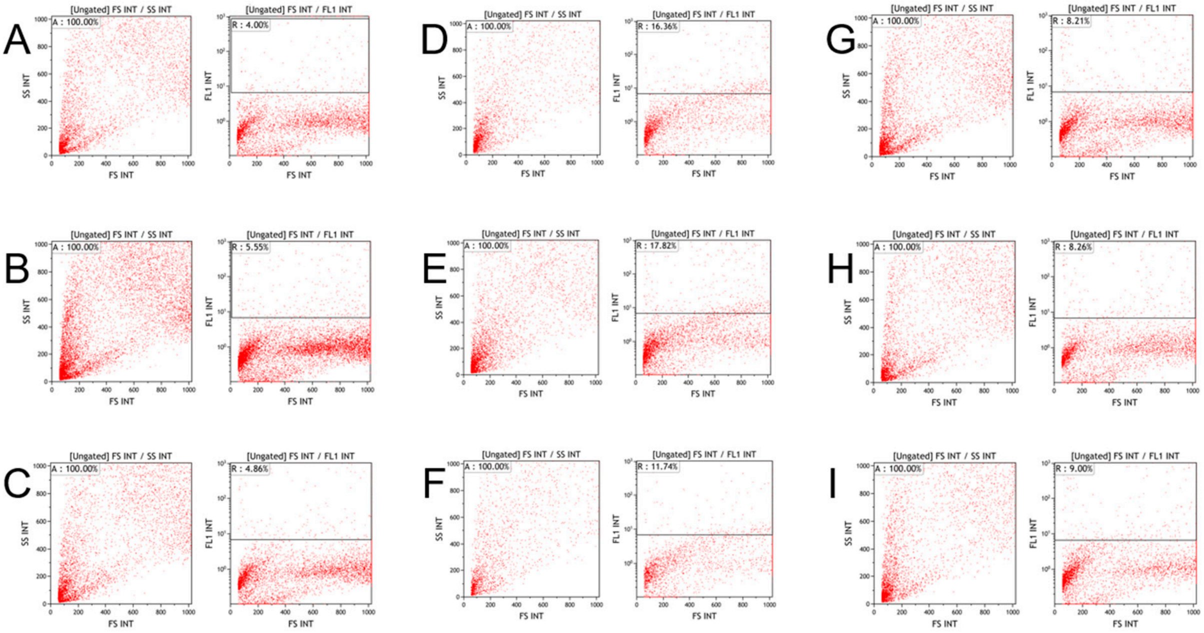

Figure S12. Characterization of SH-SY5Y cell transfection via flow cytometry. Representative dot plots display forward scatter (FS int) versus side scatter (SS int) parameters (left panels) and FS int versus FL1 fluorescence intensity (right panels) for cells transfected with (A-C) pTurboGFP-C/N_3-PEG_{114}-b-pAsp(DET)$_{62}$, (D-F) pTurboGFP-C/N_3-PEG_{114}-b-pAsp(spd)$_{62}$, (G-I) pTurboGFP-C/Dop-PEG_{114}-b-pAsp(DET)$_{62}$.
